# Geriatric Assessment in Oncology: Evolution, Evidence, and Implications for Geriatric Medicine in Japan

**DOI:** 10.1111/ggi.70433

**Published:** 2026-03-09

**Authors:** Hiroshi Yamamoto, Namio Kagoo, Ho Lee, Manami Sazuka, Chiemi Nogimori, Hirokazu Yamada, Akira Saito

**Affiliations:** ^1^ Department of Respiratory Medicine Tokyo Metropolitan Institute for Geriatrics and Gerontology Tokyo Japan

**Keywords:** cancer in older adults, comprehensive geriatric assessment, geriatric assessment, geriatric oncology, multidisciplinary care

## Abstract

This review summarizes the evolution of recommendations for geriatric assessment (GA), the randomized evidence underpinning GA‐guided management (GAM), and current implementation challenges in Japan. From the combined perspectives of oncology and geriatric medicine, we discuss how GA relates to comprehensive geriatric assessment (CGA), and argue that GA is best understood as an oncology‐adapted extension of CGA. Recent trials show that active, multidisciplinary implementation of GA is associated with improvements in treatment toxicity, functional status, communication, and quality of life. In Japan, GA remains underused because of workforce limitations and structural barriers, despite its conceptual fit with the national long‐term care insurance (LTCI) system. Progress will require practical workflows that link assessment to management, selective integration of GA with LTCI information to reduce duplication, and sustained collaboration between oncology and geriatrics. Overall, GA has moved beyond risk stratification toward a pragmatic, evidence‐supported approach that may help reframe cancer care for older adults in Japan.

## 1 Introduction

Worldwide, population aging has fundamentally transformed the epidemiology of cancer. According to official statistics, 75.6% of incident cancer cases (2021) and 88.6% of cancer deaths (2024) occurred in individuals aged 65 years or older in Japan [1]. As a result, an increasing proportion of patients present with multimorbidity, frailty, functional impairment, cognitive decline, malnutrition, polypharmacy, and limited social support. These vulnerabilities are only partly captured by the ECOG performance status [2], which describes overall functioning but does not assess key geriatric domains. As a result, older adults may be overtreated despite high vulnerability to toxicity, or undertreated despite having sufficient physiologic reserve for standard therapy.

Comprehensive geriatric assessment (CGA) originated in geriatric medicine as a structured, multidimensional, multidisciplinary evaluation designed to identify geriatric syndromes, guide interventions, and improve function and well‐being [3]. Over the past four decades, CGA has become the reference standard for evaluating older adults in clinical geriatrics, long‐term care, and rehabilitation. It involves systematic assessment across functional, cognitive, medical, psychological, nutritional, and social domains, coupled with team‐based intervention and follow‐up.

In oncology, geriatric assessment (GA) applies CGA principles to decisions around cancer treatment. GA retains the multidimensional framework of CGA while emphasizing domains most relevant to cancer therapy—functional status, comorbidity, cognition, nutrition, polypharmacy, psychological health, and social resources. GA differs from CGA less by breadth than by operational intent: CGA aims to optimize overall health and function, whereas GA aims to inform treatment selection, estimate tolerance to therapy, and reduce treatment‐related harm. Although originally deployed primarily as an assessment, recent trials increasingly evaluate GA‐linked interventions that resemble CGA‐style multidisciplinary care. GA‐guided management (GAM) refers to a care model in which vulnerabilities identified through GA are explicitly linked to structured, multidisciplinary interventions rather than being used solely for risk stratification or prognostic estimation. In this sense, GAM operationalizes GA in a manner conceptually analogous to CGA in geriatric medicine, where assessment is inseparable from subsequent management.

Accordingly, interpreting the evidence and recommendations in this review requires a clear understanding of how GA relates to CGA, particularly for readers in geriatric medicine who may be familiar with CGA but less accustomed to oncology‐specific GA frameworks.

This narrative review primarily focuses on randomized controlled trials evaluating GA–based or CGA‐like interventions in older adults with cancer, as well as major international guidelines. We prioritized trials in which GA or CGA was explicitly linked to management decisions or multidisciplinary interventions. Consistent with the 2023 American Society of Clinical Oncology (ASCO) guideline, we use the term GAM to refer to the use of GA findings both to inform cancer treatment decision‐making and to address GA‐identified impairments through appropriate interventions, counseling, and/or referrals; however, in this narrative review, our comparative synthesis primarily focuses on trials in which GA or CGA findings were operationalized into structured, multidisciplinary impairment‐directed management pathways. Accordingly, studies focusing solely on treatment selection based on GA results, or single‐center pilot interventions without structured management pathways, were not included in the main comparative framework, as the present review aims to examine the evolution of GA toward intervention‐oriented, CGA‐like care models. We also acknowledge earlier or conceptually distinct trials, such as the ESOGIA study Corre et al. [4] and a single‐center CGA intervention study Ørum et al. [5], which are briefly noted in the manuscript but were not included in the main comparative framework because their primary design focus differs from the GA‐guided, multidisciplinary management models emphasized here.

## 2 Evolution of the ASCO Guidelines

### 2.1 The 2018 ASCO Guideline

The 2018 ASCO guideline [6] formalized GA as a standard component of care for all patients aged 65 years or older receiving chemotherapy. The guidelines emphasized that GA provides clinically relevant information beyond performance status and chronological age. Functional impairment in basic activities of daily living (ADL) [7, 8] and instrumental activities of daily living (IADL) [9], comorbidity burden, cognitive impairment, malnutrition, and polypharmacy are key determinants of tolerance to chemotherapy.

The guidelines recommended incorporating prognostic tools such as ePrognosis (https://eprognosis.ucsf.edu/) and using chemotherapy toxicity prediction models including the Cancer and Aging Research Group (CARG) score [10] and the Chemotherapy Risk Assessment Scale for High‐Age Patients (CRASH) [11], both derived from CGA domains. At this stage, GA was positioned primarily as an evaluative and prognostic tool—essential to decision‐making but not yet a direct intervention.

### 2.2 The 2023 Update in the ASCO Guideline

The 2023 update in the ASCO guideline [12] signaled a major paradigm shift, redefining GA from “assessment” to “intervention.” Evidence from major randomized controlled trials (RCTs)—including GAP70+ [13] and GAIN [14]—demonstrated that GAM, in which GA findings trigger structured, tailored interventions, was associated with improved outcomes in key randomized trials. This shift is entirely consistent with the philosophy underlying CGA: assessment is meaningful only when followed by actionable, multidisciplinary management.

The updated guidelines strongly recommend GA and GAM for all older adults receiving systemic therapy, extending beyond chemotherapy to immunotherapy and targeted agents. To improve feasibility, ASCO introduced the practical geriatric assessment (PGA), a streamlined GA instrument that retains essential CGA domains while reducing the amount of time required to complete the assessment and the burden of training people to administer it.

The National Comprehensive Cancer Network (NCCN) Guideline for Older Adult Oncology also emphasizes the importance of assessing functional status, comorbidity, cognition, nutrition, and polypharmacy, although they provide less prescriptive operational guidance than the ASCO recommendations [15].

Notably, this shift toward GAM is consistent with international frameworks of geriatric oncology. In Japan, guidelines published by professional societies have similarly emphasized CGA‐based multidisciplinary care for older cancer patients, reflecting an ongoing global convergence in principles [16]. Practically, this evolution simply reflects a long‐standing clinical intuition among geriatricians: assessment is meaningful only when it drives action.

## 3 GA Within the CGA Framework: Conceptual Clarification

Because GA is derived directly from CGA, distinguishing the two while acknowledging their shared foundation is crucial for readers from the field of geriatric medicine. Although GA and CGA share common domains, they were originally developed for different purposes. CGA is a comprehensive, multidisciplinary process in geriatric medicine aimed at diagnosing geriatric syndromes, improving global health status and functional independence, and coordinating longitudinal interventions across medical, functional, cognitive, psychological, and social domains [3]. It typically involves geriatricians, nurses, rehabilitation specialists, dietitians, pharmacists, and social workers and it is embedded within hospital, ambulatory, or long‐term care systems.

In contrast, GA represents the oncology‐specific operationalization of CGA. Its primary purpose is to identify age‐related vulnerabilities that influence tolerance to treatment, risk of toxicity, and therapeutic decision‐making. The scope of GA is thus narrower in intent than CGA, focusing on the domains that are most relevant to cancer therapy. GA as an assessment does not inherently require multidisciplinary intervention; however, GAM does, and the structure of GAM increasingly mirrors the core principles of CGA. This convergence has become increasingly apparent in real‐world clinical practice.

Recent evidence suggests that the GA approaches conferring the greatest benefit are those in which GA is performed before the selection of treatment, the identified vulnerabilities lead to management recommendations that are actionable, and these interventions are delivered through multidisciplinary collaboration. Taking the above findings together, GA and CGA are best considered not as separate constructs but as points along a continuum that extends from assessment to comprehensive intervention. Clinical oncology—through trials such as GAP70+, GAIN, INTEGERATE, GERICO, and G‐oncoCOACH—is moving toward greater integration of CGA‐like, multidisciplinary care models in routine oncology practice.

## 4 Evidence From Randomized Controlled Trials

Table 1 provides a summary of the major RCTs of GA and GAM.

**TABLE 1:** Summary of major RCTs evaluating GA and GAM in older adults with cancer.

Trial, year	Country	Population	*N*	Intervention	Comparator	Primary outcome	Key results	Notes	References
Magnuson et al. 2018	USA	≥ 65 receiving chemotherapy	71	GA performed; GA summary + recommendations to oncologists	Usual care (no GA)	Feasibility; exploratory toxicity	No reduction in grade ≥ 3 toxicity	Assessment‐only; no multidisciplinary management → limited effect	[17]
Nadaraja et al., 2020	Denmark	≥ 70 with solid tumors	356	CGA performed; summary to treating oncologists	Usual oncology care	Treatment completion	No improvement in completion or toxicity	No structured GAM; limited implementation	[18]
GAP70+, 2021	USA	≥ 70 with ≥ 1 GA‐detected impairment, starting chemotherapy	718	GAM with tailored interventions (PT/OT, nutrition, medication review, ACP)	Usual care	Grade ≥ 3 toxicity	Severe toxicity reduced by ~20 percentage points; fewer falls	Landmark cluster RCT proving GAM effectiveness	[13]
GAIN, 2021	USA	Older adults starting chemotherapy	613	Nurse‐led multidisciplinary GA‐guided intervention modeled on CGA	Usual care	Grade ≥ 3 toxicity	Reduced toxicity; more AD; improved medication management	Explicit CGA‐like team model; high fidelity	[14]
INTEGERATE, 2022	Australia	Older adults starting systemic therapy	154	Integrated geriatric–oncology care before and during therapy	Usual oncology care	HRQOL	Improved physical function; fewer unplanned admissions	Full geriatric–oncology collaborative care	[19]
GERICO, 2021	Denmark	Frail older adults with colorectal cancer	160	CGA‐based interventions (rehab, nutrition, medication review)	Usual care	Treatment completion	Improved mobility; higher chemotherapy completion	High‐risk population; strong rehab component	[20]
PROGNOSIS‐RCT, 2024	Switzerland	Mildly frail older adults with advanced cancer	335	Multidimensional CGA‐guided plan	Standard oncology care	Physical performance	No significant benefit overall; borderline effect in least frail subgroup	Effect seen even in mildly frail patients	[21]
5C Trial, 2023	Canada	Older adults receiving cancer treatment	350	GA + management recommendations	Standard care	QOL	No overall QOL benefit; post hoc suggests early GA → more benefit	Important trial on GA timing	[22]
COACH, 2020	USA	≥ 70 undergoing treatment	541	GA used to structure oncologist–patient–caregiver communication	Usual communication	Communication quality	Improved communication; better shared decision‐making	Focused on communication outcomes	[23]
G‐oncoCOACH, 2025	Belgium	Older adults receiving systemic therapy in high‐GA‐use system	430	CGA + intensive patient coaching	GA‐based care	QOL	Significant QOL improvement despite already high baseline GA use	Shows added benefit of patient activation + coaching	[24]

Abbreviations: AD, advance directive; CGA, comprehensive geriatric assessment; GA, geriatric assessment; GAM, GA‐guided management; HRQOL, health‐related quality of life; PT/OT, physical therapy/occupational therapy; QOL, quality of life; RCT, randomized controlled trial.

### 4.1 Early Trials: Assessment Alone Is Insufficient

Early pilot trials (Magnuson [17], Nadaraja [18]) evaluated GA without structured intervention. Providing GA summaries to oncologists or multidisciplinary teams did not reduce toxicity or improve the rate of treatment completion, underscoring that assessment without intervention has limited impact—a finding consistent with the long‐held principle within the field of geriatric medicine that CGA must be paired with action to be effective.

### 4.2 Reduced Chemotherapy Toxicity Through GAM

GAP70+ [13] demonstrated that GAM significantly reduced the incidence of severe chemotherapy‐related toxicity and falls. The intervention consisted of providing oncologists with a tailored GA summary and management recommendations addressing aging‐related vulnerabilities identified through GA, with guidance informed by geriatric principles and consensus‐based recommendations.

GAIN [14] further demonstrated that a geriatrics‐trained, multidisciplinary GA‐driven intervention—coordinated primarily by nurse practitioners—reduced chemotherapy‐related toxicity and significantly increased advance directive completion. These findings support the notion that the clinical benefits of GA are realized when assessment is coupled with structured, multidisciplinary intervention consistent with CGA principles.

### 4.3 Improvements in Quality of Life (QOL), Function, and Treatment Completion

INTEGERATE [19] demonstrated that CGA integrated into oncology care led to better health‐related quality of life (HRQOL) over 24 weeks (measured by the Elderly Functional Index), fewer unplanned hospital admissions by 24 weeks, and higher chemotherapy completion, while overall survival was not significantly different between groups.

In GERICO [20], among vulnerable older patients with colorectal cancer (screened as frail/vulnerable), a CGA‐based intervention increased completion of planned chemotherapy without further dose reductions or delays (45% vs. 28%) and was associated with fewer secondary dose reductions and a higher proportion receiving full‐dose chemotherapy in all cycles; grade ≥ 3 toxicity and hospitalization were numerically improved or similar but not statistically different. Importantly, these findings indicate that GA does not simply function as a tool for treatment de‐escalation but also plays a critical role in identifying older patients who can tolerate and benefit from adequate‐intensity therapy, thereby preventing inappropriate undertreatment.

By contrast, PROGNOSIS‐RCT [21]—testing CGA‐guided interventions added to standard oncologic care in frail older adults initiating palliative treatment—did not show statistically significant between‐group differences in the primary endpoint (30 s chair stand test at 3 months) or in quality of life (QOL) measures overall, although subgroup analyses suggested a borderline improvement in physical performance among the least frail participants.

Finally, the 5C trial [22] found no effect of GA and management on the primary outcome of global QOL or on other endpoints including overall survival, treatment plan modification, toxicity, unplanned hospitalizations, or emergency department visits. In discussing potential reasons for these null findings, the authors note that many participants completed GA on the day of their first treatment, which may have limited GA‐triggered changes to the oncologic plan, and they state that GA‐based management completed before the final treatment decision is most effective.

### 4.4 Communication‐Focused Outcomes

The COACH trial [23] demonstrated that provision of a structured GA summary with GA‐guided recommendations to community oncologists significantly improved patient‐ and caregiver‐centered communication regarding aging‐related concerns. Compared with usual care, the intervention increased patient satisfaction with communication about aging‐related issues and sustained this improvement over 6 months. It also markedly increased both the number and quality of conversations addressing geriatric domains, as well as discussions that led to GA‐driven recommendations.

Notably, COACH did not demonstrate improvements in QOL or survival, underscoring that its primary impact was on care processes rather than downstream clinical outcomes. Nevertheless, by facilitating recognition and discussion of functional, cognitive, and psychosocial vulnerabilities that are often overlooked in routine oncology practice, COACH provides robust evidence that GA can operationalize core CGA principles—namely, patient‐centered communication and shared decision‐making—even in community oncology settings without direct geriatrician involvement.

### 4.5 Evidence From a High‐Implementation Setting: The G‐oncoCOACH Trial

The G‐oncoCOACH trial [24] provided compelling evidence that the effectiveness of GA depends not only on assessment but also on active implementation and patient engagement. Conducted in Belgium, where GA is already routinely performed, this study showed that integrating a geriatric team to implement GA‐based recommendations, combined with intensive patient coaching, resulted in a clinically meaningful improvement in QOL. The contrast between the intervention and control groups—both of which underwent GA—highlights that structured implementation and patient activation are essential to translating assessment into improved outcomes.

### 4.6 Integrated Interpretation Across Trials

To better understand why some randomized trials demonstrated clear benefits whereas others yielded neutral results, it is useful to interpret the evidence along several key dimensions: (1) whether GA was linked to active intervention or remained assessment‐only, (2) the timing of GA in relation to treatment decision‐making, (3) the intensity and fidelity of multidisciplinary implementation, and (4) the characteristics of the target population and selected endpoints.

Across these randomized controlled trials, several consistent but nuanced themes emerge regarding the role of GA in oncology. First, trials evaluating GA as an assessment alone, without structured implementation, consistently demonstrate limited clinical impact. In contrast, benefits in specific clinical outcomes are observed when GA findings are translated into actionable, individualized management strategies that address specific vulnerabilities identified through assessment.

Second, the outcomes influenced by GAM vary across trials, reflecting differences in study populations, intervention intensity, and primary endpoints. While reductions in chemotherapy‐related toxicity were clearly demonstrated in trials such as GAP70+ and GAIN, other studies highlighted improvements in physical function, QOL, treatment completion, unplanned hospitalizations, or communication quality. Importantly, these outcomes were not uniformly observed across all trials, underscoring that the effect of GA is context‐dependent rather than universal.

Third, the timing and mode of GA implementation appear to be critical determinants of effectiveness. Evidence from trials such as 5C suggests that GA performed after treatment decisions may have limited impact, whereas studies integrating GA earlier in the treatment pathway were more likely to demonstrate benefits of practical clinical relevance. This observation aligns closely with principles long emphasized in CGA, in which anticipatory, rather than reactive, intervention is central.

Fourth, across positive trials, the involvement of multiple health‐care professionals—including geriatricians, nurses, rehabilitation specialists, dietitians, pharmacists, and social workers—emerged as a common feature of effective interventions. Studies that embedded geriatric expertise within oncology care pathways, rather than merely reporting GA findings to oncologists, were more likely to achieve improvements in patient‐centered outcomes.

Finally, recent trials such as G‐oncoCOACH further indicate that patient activation and structured follow‐up are important components of successful GA‐based care. Even in settings where GA is already routinely performed, additional emphasis on implementation fidelity and patient engagement is required to translate assessment into improved QOL.

Taken together, these findings indicate that the most effective models of GAM are not defined by assessment alone, but by how assessment is operationalized within clinical care. Although GA originated as an oncology‐specific adaptation of CGA, contemporary evidence suggests that its clinical impact increasingly depends on adopting core CGA principles, including multidimensional assessment, interdisciplinary collaboration, and active implementation of individualized care plans. In this sense, rather than representing a departure from CGA, modern GAM can be viewed as an oncology‐contextualized extension of comprehensive geriatric intervention.

## 5 Implementation Challenges and Opportunities in Japan

In Japan, an important step toward CGA‐based oncology care has recently been taken. Specifically, in 2024, the Japan Geriatrics Society published the Clinical Practice Guideline for Care Based on Comprehensive Geriatric Assessment (2024), which strongly recommended (recommendation level: Grade 1A) that CGA be performed for older adults undergoing cancer pharmacotherapy [16]. Although these are guidelines published by a medical society rather than by the Japanese government, this signals a clear direction toward evidence‐informed geriatric care in Japan and is well aligned with the country's aging demographics and long‐term care infrastructure.

These developments provide a strong foundation for the implementation of GA and GAM within oncology practice in Japan. However, there are still substantial gaps between recommendations in guidelines and real‐world practice, particularly in workflow integration, staffing, and cross‐sector coordination. To date, GA has only been implemented to a limited extent in Japan. Indeed, a national mixed‐methods study found that GA is not routinely performed in almost 80% of oncology facilities [25], with the factors proposed as explaining this including insufficient knowledge, limited staffing, and the absence of structured pathways.

### 5.1 System‐Level and Structural Barriers

Notably, several of the above‐mentioned barriers have been repeatedly described in Japanese oncology settings. Qualitative research based on the CFIR framework [26] revealed that implementation depends more on organizational readiness and workflow integration than on the individual effort of clinicians. These findings align with the BeST‐GA scoping review [27], which identified similar structural barriers internationally.

### 5.2 Integration With Japan's Long‐Term Care Insurance (LTCI) System

Japan's LTCI system is a mandatory, nationwide public insurance program that provides needs‐based certification and services for older adults requiring support or care. The LTCI assessment process routinely evaluates functional status, cognitive function, and social support, resulting in standardized care‐level certification that closely overlaps with key CGA domains.

Japan's LTCI system conducts functional, cognitive, and social assessments that parallel CGA domains. Approximately one‐quarter of physicians already use LTCI results when selecting cancer treatment [25]. Integrating GA with LTCI could reduce duplication and provide a CGA‐informed platform, even in cancer centers where resources are limited.

### 5.3 Necessity of Multidisciplinary Collaboration

It is important to note that effective GAM inherently depends on interdisciplinary collaboration, which is a defining feature of CGA. Many of the interventions shown to improve outcomes in RCTs—such as medication review, nutritional support, physical rehabilitation, cognitive assessment, and social care coordination—are not procedures that oncologists can perform alone. Rather, they require coordinated input from pharmacists, dietitians, rehabilitation specialists, nurses, and social workers. This structure, which is consistent with CGA, explains why early trials relying solely on providing GA summaries without active multidisciplinary implementation failed to achieve clinical benefit [17, 18].

More recent trials—including GAIN, INTEGERATE, GERICO, and G‐oncoCOACH—further reinforce the assertion that GA improves outcomes only when its findings are translated into coordinated, multidisciplinary interventions [19, 20, 24]. These studies collectively demonstrate that the effectiveness of GAM arises not from the assessment itself but from the CGA‐like team‐based implementation that follows it.

### 5.4 Toward a Hybrid Implementation Model for Japan

Given the diversity of clinical settings and workforce constraints across Japan, a hybrid implementation model that builds on validated domestic tools and existing care infrastructure may offer the most feasible and scalable approach to expanding GA and GAM. Although the 2023 ASCO guideline introduced the PGA as a streamlined tool to support feasibility, PGA has not been linguistically or psychometrically validated in Japan and should not be adopted as a first‐line screening strategy in its current form. Instead, initial screening should rely on instruments that have been validated in older Japanese populations—such as the G8 screening tool (G8) [28], Vulnerable Elders Survey‐13 (VES‐13) [29], the Kihon Checklist (KCL) [30], or institution‐specific GA instruments—followed by full GA or CGA for patients identified as high‐risk.

Conceptually, this hybrid implementation model can be understood as a dual‐pathway framework: (1) a direct GA/CGA‐driven multidisciplinary care pathway, and (2) an LTCI‐linked pathway that can support assessment, intervention, and care coordination in settings where full geriatric multidisciplinary implementation is not feasible. Incorporating these validated screening tools into routine oncology workflows can help identify patients who would benefit the most from comprehensive, CGA‐based interventions. For individuals with substantial vulnerabilities, a full GA or CGA can then be conducted, enabling tailored management plans aligned with the principles demonstrated in successful RCTs. Importantly, because many GAM components—such as medication review, nutritional support, rehabilitation, and social care coordination—require interdisciplinary expertise, nurse‐led or allied health–led pathways might facilitate implementation while maintaining fidelity to the CGA framework.

Japan's LTCI system provides an additional unique opportunity for integration. LTCI assessments capture functional, cognitive, and social domains that overlap substantially with GA and CGA, and their incorporation into oncology workflows can reduce duplication, improve efficiency, and enhance coordination with community‐based geriatric services. A hybrid model that links validated screening tools, GA/CGA for high‐risk patients, and LTCI information may therefore facilitate a sustainable, context‐appropriate expansion of GA/CGA‐informed oncology care in Japan.

**FIGURE 1:**
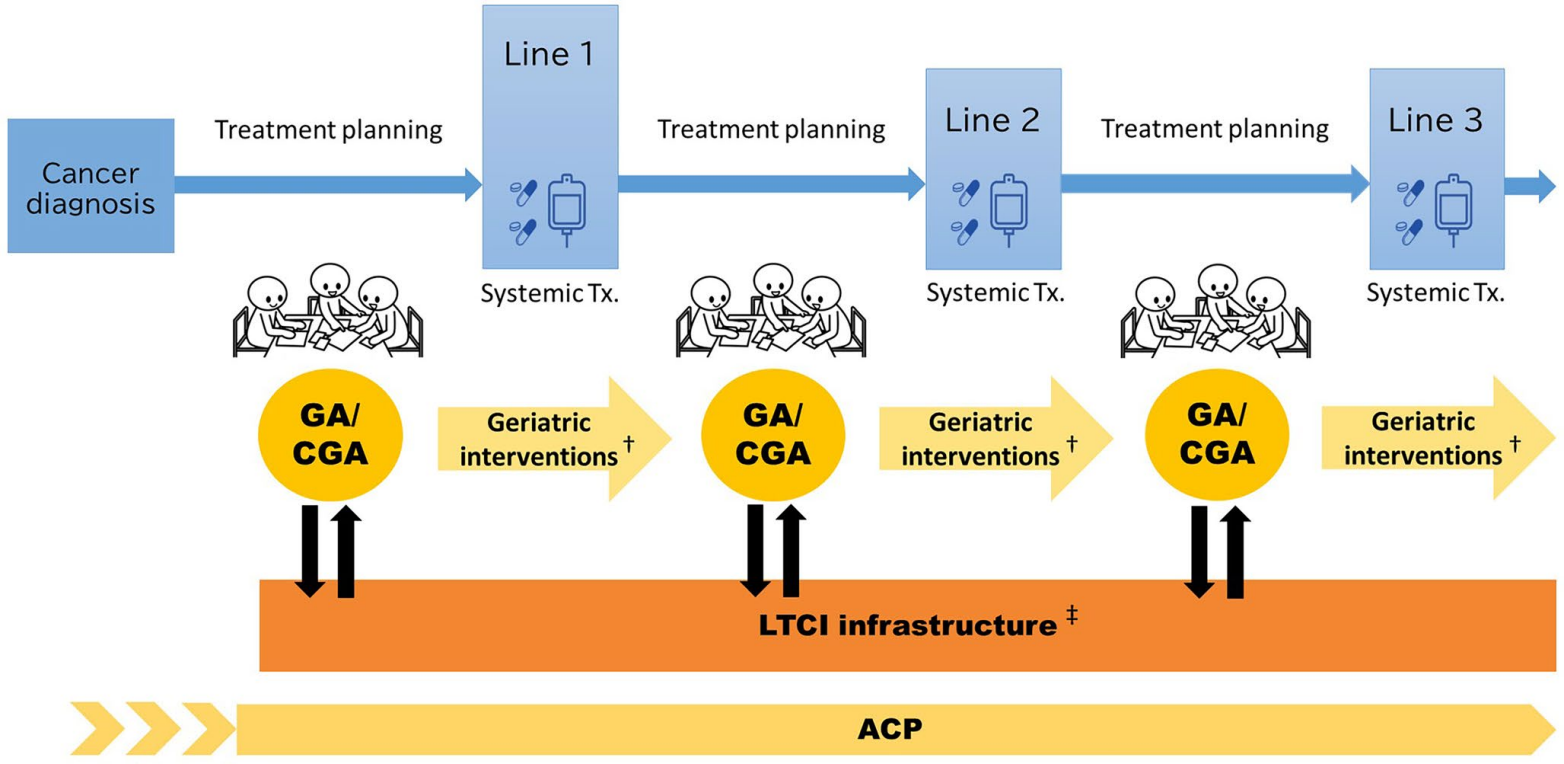
A practical hybrid implementation model for GA/CGA‐guided care in older adults with cancer integrating the Japanese LTCI system. GA/CGA is performed before each treatment line, interpreted by a multidisciplinary team, and translated into geriatric interventions. In Japan, the LTCI system functions as an implementation infrastructure supporting assessment, intervention, and care coordination across the disease trajectory, even when geriatric multidisciplinary implementation capacity is limited. ACP underpins the entire care process. † MDT interpretation and care planning. ‡ The LTCI system functions as an implementation infrastructure supporting assessment, intervention, and care coordination. Even when CGA‐based multidisciplinary implementation cannot be fully provided, LTCI linkage can partially substitute implementation capacity. ACP, advance care planning; CGA, comprehensive geriatric assessment; GA, geriatric assessment; LTCI, long‐term care insurance; MDT, multidisciplinary team; Tx, treatment.

Operationally, this model assumes a stepwise workflow embedded in routine oncology practice rather than a parallel geriatric program. For example, in routine practice, LTCI certification status and care‐level information could be reviewed at the time of oncology referral, followed by brief screening using tools such as the G8 or KCL. Patients identified as high‐risk could then undergo focused GA or CGA, with predefined referral pathways to rehabilitation, nutrition, pharmacy, and social work services. In such a model, nurse‐led or allied health–led coordination, supported by geriatricians, could enable scalable implementation even in resource‐limited settings (Figure 1).

## 6 Future Directions

To ensure progress of GA in Japan, various practical priorities require attention. First, standardized workflows that reliably connect GA findings to CGA‐like multidisciplinary interventions are essential to achieve consistent effects in routine clinical practice. Second, digital infrastructure should be developed to automate the administration of GA, streamline data capture, and facilitate integration with electronic medical records. Third, education and training programs must strengthen GA and CGA literacy across oncology teams, enabling clinicians to interpret results and operationalize recommended interventions. Fourth, collaboration that makes use of Japan's LTCI system should be expanded to improve coordination between oncology services and community‐based geriatric care. Finally, policy reform is needed to formally recognize and appropriately reimburse CGA‐based oncologic care, thereby supporting sustainable implementation at scale.

## 7 Conclusion

GA, derived from and grounded in the principles of CGA, has evolved from a tool for risk stratification into an evidence‐based approach that can improve treatment safety, functional outcomes, communication, and QOL for older adults with cancer. Evidence from randomized controlled trials consistently demonstrates that GA achieves its greatest impact not when confined to assessment alone, but when it is translated into structured, individualized interventions delivered through multidisciplinary care.

Importantly, GA must not be regarded as a static evaluation performed at the time of treatment initiation. Rather, the available evidence indicates that GA is most effective when conducted before final treatment decisions are made, allowing identified vulnerabilities to meaningfully inform therapeutic planning and supportive care strategies. Trials in which GA was performed only after treatment initiation, or in which implementation of GA‐based recommendations was limited, have shown attenuated or null effects, underscoring the importance of timing and implementation fidelity.

Across studies demonstrating positive outcomes, a common feature is the active translation of GA findings into coordinated management, with geriatric expertise playing a central role in guiding assessment, prioritizing vulnerabilities, and facilitating appropriate interventions. Although GA originated as an oncology‐adapted framework, its clinical effectiveness increasingly depends on adopting core CGA principles, including anticipatory assessment, interdisciplinary collaboration, and proactive intervention rather than reactive management.

In Japan, integrating GA‐ and CGA‐based frameworks across oncology and long‐term care systems remains a critical challenge. Addressing this challenge will require system‐level approaches, team‐based workflows, and deliberate alignment with the national LTCI structure. The broader adoption of GA thus represents not merely a technical addition to oncology practice, but a necessary step toward individualized, value‐based cancer care in a rapidly aging society.

Ultimately, GA should not be viewed as separate from CGA, but rather as its oncology‐adapted extension. Contemporary evidence suggests that the true value of GA lies in its capacity to operationalize long‐established geriatric principles within cancer care—principles that emphasize early assessment, meaningful intervention, and multidisciplinary collaboration. For Japan, embracing this perspective offers an opportunity to reposition geriatric medicine at the core of oncology practice and to advance truly person‐centered, geriatric‐informed cancer care.

## Author Contributions

Hiroshi Yamamoto conceptualized the review topic, designed the overall structure, and drafted the initial manuscript. Hiroshi Yamamoto led the integration of oncology and geriatric medicine perspectives and critically revised all sections of the manuscript for intellectual content. Namio Kagoo, Ho Lee, Manami Sazuka, Chiemi Nogimori, Hirokazu Yamada, and Akira Saito contributed to the literature review, interpretation of evidence, and provided critical revisions to the manuscript. All authors reviewed and approved the final version of the manuscript.

## Funding

The authors have nothing to report.

## Disclosure

Hiroshi Yamamoto has received lecture fees from AstraZeneca K.K., Chugai Pharmaceutical Co. Ltd., Bristol‐Myers Squibb K.K., Taiho Pharmaceutical Co. Ltd., Boehringer Ingelheim Japan, K.K., Nippon Kayaku Co. Ltd., Kyowa Kirin Co. Ltd., MSD K.K., Daiichi Sankyo Co. Ltd., Teijin Healthcare Co. Ltd., Sanofi K.K., Insmed G.K., Life Technologies Japan Ltd., Kyorin Pharmaceutical Co. Ltd., and GlaxoSmithKline K.K. within the past 36 months. Akira Saito has received lecture fees from Taiho Pharmaceutical Co. Ltd.

## Ethics Statement

The authors have nothing to report.

## Consent

The authors have nothing to report.

## Conflicts of Interest

The authors declare no conflicts of interest.

## Data Availability

Data sharing is not applicable to this article as no datasets were generated or analyzed during the current study.

## References

[1] Cancer Registry and Statistics, Cancer Information Service NCC, Japan , “Cancer Statistics in Japan; Table Download,” Website Cancer Statistics in Japan; Table Download, https://ganjoho.jp/reg_stat/statistics/data/dl/index.html.

[2] M. M. Oken , R. H. Creech , D. C. Tormey , et al., “Toxicity and Response Criteria of the Eastern Cooperative Oncology Group,” American Journal of Clinical Oncology 5 (1982): 649–655. 7165009

[3] A. E. Stuck , A. L. Siu , G. D. Wieland , J. Adams , and L. Z. Rubenstein , “Comprehensive Geriatric Assessment: A Meta‐Analysis of Controlled Trials,” Lancet 342 (1993): 1032–1036, 10.1016/0140-6736(93)92884-v. 8105269

[4] R. Corre , L. Greillier , H. Le Caër , et al., “Use of a Comprehensive Geriatric Assessment for the Management of Elderly Patients With Advanced Non‐Small‐Cell Lung Cancer: The Phase III Randomized ESOGIA‐GFPC‐GECP 08‐02 Study,” Journal of Clinical Oncology 34 (2016): 1476–1483, 10.1200/jco.2015.63.5839. 26884557

[5] M. Ørum , S. V. Eriksen , M. Gregersen , et al., “The Impact of a Tailored Follow‐Up Intervention on Comprehensive Geriatric Assessment in Older Patients With Cancer—A Randomised Controlled Trial,” Journal of Geriatric Oncology 12 (2021): 41–48, 10.1016/j.jgo.2020.07.011. 32747208

[6] S. G. Mohile , W. Dale , M. R. Somerfield , et al., “Practical Assessment and Management of Vulnerabilities in Older Patients Receiving Chemotherapy: ASCO Guideline for Geriatric Oncology,” Journal of Clinical Oncology 36 (2018): 2326–2347, 10.1200/jco.2018.78.8687. 29782209 PMC6063790

[7] S. Katz , “Assessing Self‐Maintenance: Activities of Daily Living, Mobility, and Instrumental Activities of Daily Living,” Journal of the American Geriatrics Society 31 (1983): 721–727. 6418786 10.1111/j.1532-5415.1983.tb03391.x

[8] F. I. Mahoney and D. W. Barthel , “Functional Evaluation: The Barthel Index,” Maryland State Medical Journal 14 (1965): 61–65. 14258950

[9] M. P. Lawton and E. M. Brody , “Assessment of Older People: Self‐Maintaining and Instrumental Activities of Daily Living,” Gerontologist 9 (1969): 179–186. 5349366

[10] A. Hurria , K. Togawa , S. G. Mohile , et al., “Predicting Chemotherapy Toxicity in Older Adults With Cancer: A Prospective Multicenter Study,” Journal of Clinical Oncology 29 (2011): 3457–3465, 10.1200/JCO.2011.34.7625. 21810685 PMC3624700

[11] M. Extermann , I. Boler , R. R. Reich , et al., “Predicting the Risk of Chemotherapy Toxicity in Older Patients: The Chemotherapy Risk Assessment Scale for High‐Age Patients (CRASH) Score,” Cancer 118 (2012): 3377–3386, 10.1002/cncr.26646. 22072065

[12] W. Dale , H. D. Klepin , G. R. Williams , et al., “Practical Assessment and Management of Vulnerabilities in Older Patients Receiving Systemic Cancer Therapy: ASCO Guideline Update,” Journal of Clinical Oncology 41 (2023): 4293–4312, 10.1200/jco.23.00933. 37459573 PMC12803700

[13] S. G. Mohile , M. R. Mohamed , H. Xu , et al., “Evaluation of Geriatric Assessment and Management on the Toxic Effects of Cancer Treatment (GAP70+): A Cluster‐Randomised Study,” Lancet 398 (2021): 1894–1904, 10.1016/s0140-6736(21)01789-x. 34741815 PMC8647163

[14] D. Li , C. L. Sun , H. Kim , et al., “Geriatric Assessment‐Driven Intervention (GAIN) on Chemotherapy‐Related Toxic Effects in Older Adults With Cancer: A Randomized Clinical Trial,” JAMA Oncology 7 (2021): e214158, 10.1001/jamaoncol.2021.4158. 34591080 PMC8485211

[15] National Comprehensive Cancer Network (NCCN) , “Website Older Adult Oncology (Version 2. 2025), Older Adult Oncology (Version 2.),” 2025, https://www.nccn.org/professionals/physician_gls/pdf/senior.pdf.

[16] Japan Geriatrics Society , Clinical Practice Guidelines for Care Based on Comprehensive Geriatric Assessment (2024) (Nanzando, 2024).

[17] A. Magnuson , T. Lemelman , C. Pandya , et al., “Geriatric Assessment With Management Intervention in Older Adults With Cancer: A Randomized Pilot Study,” Support Care Cancer 26 (2018): 605–613, 10.1007/s00520-017-3874-6. 28914366 PMC5887127

[18] S. Nadaraja , L. E. Matzen , T. L. Jørgensen , et al., “The Impact of Comprehensive Geriatric Assessment for Optimal Treatment of Older Patients With Cancer: A Randomized Parallel‐Group Clinical Trial,” Journal of Geriatric Oncology 11 (2020): 488–495, 10.1016/j.jgo.2019.06.019. 31279749

[19] W. K. Soo , M. T. King , A. Pope , P. Parente , P. Dārziņš , and I. D. Davis , “Integrated Geriatric Assessment and Treatment Effectiveness (INTEGERATE) in Older People With Cancer Starting Systemic Anticancer Treatment in Australia: A Multicentre, Open‐Label, Randomised Controlled Trial,” Lancet Healthy Longevity 3 (2022): e617–e627, 10.1016/s2666-7568(22)00169-6. 36102776

[20] C. M. Lund , K. K. Vistisen , A. P. Olsen , et al., “The Effect of Geriatric Intervention in Frail Older Patients Receiving Chemotherapy for Colorectal Cancer: A Randomised Trial (GERICO),” British Journal of Cancer 124 (2021): 1949–1958, 10.1038/s41416-021-01367-0. 33828260 PMC8185087

[21] A. W. Giger , H. M. Ditzel , H. J. Ditzel , et al., “Effects of Comprehensive Geriatric Assessment‐Guided Interventions on Physical Performance and Quality of Life in Older Patients With Advanced Cancer: A Randomized Controlled Trial (PROGNOSIS‐RCT),” Journal of Geriatric Oncology 15 (2024): 101658, 10.1016/j.jgo.2023.101658. 37939628

[22] M. Puts , N. Alqurini , F. Strohschein , et al., “Impact of Geriatric Assessment and Management on Quality of Life, Unplanned Hospitalizations, Toxicity, and Survival for Older Adults With Cancer: The Randomized 5C Trial,” Journal of Clinical Oncology 41 (2023): 847–858, 10.1200/jco.22.01007. 36473126 PMC9902020

[23] S. G. Mohile , R. M. Epstein , A. Hurria , et al., “Communication With Older Patients With Cancer Using Geriatric Assessment: A Cluster‐Randomized Clinical Trial From the National Cancer Institute Community Oncology Research Program,” JAMA Oncology 6 (2020): 196–204, 10.1001/jamaoncol.2019.4728. 31697365 PMC6865234

[24] C. Kenis , L. Peeters , L. Laethem , et al., “Effectiveness of Comprehensive Geriatric Assessment With Extensive Patient Coaching for Improving Quality of Life in Older Patients With Solid Tumours Receiving Systemic Therapy (G‐oncoCOACH): A Multicentre Randomised Controlled Trial,” Lancet Healthy Longevity 6 (2025): 100743, 10.1016/j.lanhl.2025.100743. 40818480

[25] Y. Yoshida and K. Tamura , “Implementation of Geriatric Assessment and Long‐Term Care Insurance System by Medical Professionals in Cancer Treatment: A Nationwide Survey in Japan,” Japanese Journal of Clinical Oncology 52 (2022): 449–455, 10.1093/jjco/hyac020. 35199168

[26] A. Matsuoka , T. Mizutani , Y. Kaji , et al., “Barriers and Facilitators to Implementing Geriatric Assessment in Daily Oncology Practice in Japan: A Qualitative Study Using an Implementation Framework,” Journal of Geriatric Oncology 14 (2023): 101625, 10.1016/j.jgo.2023.101625. 37708801

[27] C. Y. Han , O. A. Agbejule , L. Kasherman , et al., “Geriatric Assessment in Older Adults With Cancer: A Global Scoping Review of Barriers and Strategies to Implementation,” Journal of Geriatric Oncology 16 (2025): 102347, 10.1016/j.jgo.2025.102347. 40845619

[28] C. A. Bellera , M. Rainfray , S. Mathoulin‐Pélissier , et al., “Screening Older Cancer Patients: First Evaluation of the G‐8 Geriatric Screening Tool,” Annals of Oncology 23 (2012): 2166–2172, 10.1093/annonc/mdr587. 22250183

[29] D. Saliba , M. Elliott , L. Z. Rubenstein , et al., “The Vulnerable Elders Survey: A Tool for Identifying Vulnerable Older People in the Community,” Journal of the American Geriatrics Society 49 (2001): 1691–1699, 10.1046/j.1532-5415.2001.49281.x. 11844005

[30] S. Satake , K. Senda , Y. J. Hong , et al., “Validity of the Kihon Checklist for Assessing Frailty Status,” Geriatrics & Gerontology International 16 (2016): 709–715, 10.1111/ggi.12543. 26171645

